# Tissue Inhibitor of Metalloproteinase 1 Is Preferentially Expressed in Th1 and Th17 T-Helper Cell Subsets and Is a Direct Stat Target Gene

**DOI:** 10.1371/journal.pone.0059367

**Published:** 2013-03-26

**Authors:** Adewole Adamson, Kamran Ghoreschi, Matthew Rittler, Qian Chen, Hong-Wei Sun, Golnaz Vahedi, Yuka Kanno, William G. Stetler-Stevenson, John J. O’Shea, Arian Laurence

**Affiliations:** 1 Molecular Immunology and Inflammation Branch, National Institute of Arthritis, Musculoskeletal and Skin Diseases, National Institutes of Health, Bethesda, Maryland, United States of America; 2 Radiation Oncology Branch, National Cancer Institute, Bethesda, Maryland, United States of America; 3 Division of Gastroenterology, Department of Internal Medicine, Tongji Medical College, Huazhong University of Science and Technology, Wuhan, Hubei Province, China; 4 Harvard-Massachusetts Institute of Technology Division of Health, Sciences and Technology, Boston, Massachusetts, United States of America; 5 Department of Dermatology, University Medical Center, Eberhard Karls University, Tübingen, Germany; Leiden University Medical Center, The Netherlands

## Abstract

CD4^+^ T helper (Th) cells differentiate into distinct effector subsets that are critical for host defense, but are also implicated in the pathogenesis of autoimmune disorders. Thelper17 (Th17) cells in particular are emerging as important drivers of multiple diseases including psoriasis, spondyloarthropathy and multiple sclerosis. To gain insight into the function of Th17 cells, we performed transcriptional profiling in hopes of elucidating products not previously recognized as being functionally relevant in these T cells. Herein, we demonstrate that tissue inhibitor of metalloproteinase 1 (TIMP1), a secreted protein with pleiotropic effects on cellular growth, survival and integrity of the extracellular matrix, is preferentially produced by Th17 and Th1 cells. We further show that Th1 and Th17 cell TIMP1 regulation follows separate mechanisms with a requirement for STAT4 in the former and STAT3 in the latter. Finally, we demonstrate that when restricted to T cells, expression of TIMP1 promotes neuropathology in experimental allergic encephalomyelitis.

## Introduction

Following engagement of the T-cell receptor, naïve CD4^+^ T cells follow distinct developmental pathways that result in the generation of distinct subsets, which are defined by the cytokines they preferentially express [1]. Differentiated helper T (Th) cells are central players in the adaptive immune response. They facilitate the effective elimination of pathogenic micro-organisms but conversely are also involved in the pathogenesis of many autoimmune diseases. Classically these cells have been viewed as being in either of two distinct subsets, Th1 or Th2. The former produce IFN-γ and are critical for host defense against intracellular pathogens including viral infections and tuberculosis [2]. Historically, Th1 cells have been suggested to be major drivers of autoimmune diseases such as experimental autoimmune encephalomyelitis (EAE) [3]. In such disorders, high levels of IFN-γ and large proportions of IFN-γ-producing cells are present. In contrast Th2 cells are required for host defense against helminth infections and have been implicated in the development of atopic disease and asthma through the production of IL-4 and IL-13 [4].

In EAE however, the role of IFN-γ and its inducing factor, IL-12 were appreciated to be more complex [5]. This led to the recognition of T cells that selectively produce IL-17, IL-21 and IL-22. These cells, denoted Th17 cells, are now recognized to be involved in the pathogenesis of mouse models of autoimmune diseases including EAE and collagen-induced arthritis [6]–[8]. Th17 cells have been implicated in human autoimmune disease including rheumatoid arthritis, ankylosing spondylitis, inflammatory bowel disease, and psoriasis [9]–[12]. Furthermore, there is emerging genetic evidence that link pathways regulating IL-17 with human disease [13]–[15].

In effort to gain further insight into potentially pathogenic Th1 and Th17 cells and the cytokines that they are able to secrete when activated, we performed transcriptional profiling to try to define new features of these subsets. We found that functionally active tissue inhibitor of metalloproteinase 1 (TIMP1), is highly expressed in both Th17 and Th1 cells. We further show that the gene encoding TIMP1 is a direct STAT target gene. Furthermore, T cell derived TIMP1, a factor implicated in autoimmune and atopic disease, promotes immune-mediated neuropathology.

## Materials and Methods

### Ethics Statement

All mice were housed under specific pathogen-free conditions. Experiments were performed in accordance with the guidelines of the NIH Animal Care and Use Committee. The protocol was approved by the NIAMS animal care and use committee (Protocol number: A011-04-01).

### Mice

Mice bearing loxP-flanked conditional (fl/fl) alleles of STAT3 on a C57BL/6J inbred background have been previously described [16] were kindly given by D. Levy (New York university). *Stat3^fl/fl^* mice were bred with mice expressing Cre under the control of the CD4 promoter (CD4-Cre) to produce *Stat3^fl/fl^;CD4-Cre* mice. *Stat4^−/−^* on a C57BL/6J background, were a kind gift from M.H. Kaplan (Indiana University) [17]. 2D2 mice and *Timp1^−/−^* were obtained from Jackson laboratories and bred to produce *Timp1^−/−^*2D2 animals. All animals were handled and housed in accordance with the guidelines of the NIH Animal Care and Use Committee. The protocol was approved by the NIAMS animal care and use committee (Protocol number: A011-04-01).

### Isolation of Cells and Cell Culture

Naïve (CD4^+^ CD62L^+^ CD44**^−^** CD25**^−^**) T cells were isolated by disrupting spleens of 6- to 12-week-old mice that had been euthanized with CO_2_. Unless stated otherwise, all cell cultures were performed in RPMI supplemented with 10% fetal calf serum, 2 mM glutamine, 100 iu/mL penicillin, 0.1 mg/mL streptomycin (Invitrogen), and 2 µM β-mercaptoethanol. T cells were enriched with a Mouse CD4^+^ T Cell Isolation Kit with an autoMacs (Miltenyi Biotec, Bergisch Gladbach, Germany). Naive T cells were obtained by surface staining with anti-CD4, anti-CD62L, anti-CD44, and anti-CD25 (BD Bioscience, San Jose, CA). The CD4^+^CD62L^+^CD44**^−^**CD25**^−^** population was isolated by flow cytometry cell sorting with a Mo-Flo cell sorter (Dako Carpinteria, CA). Cells were activated by plate-bound anti-CD3 (5 µg/mL) and soluble anti-CD28 (5 µg/mL) (BioXCell, West Lebanon, NH) for 3 days (unless otherwise indicated) and cultured either under neutral conditions with no exogenous cytokines and anti-cytokine antibodies (Th0 conditions) or in the presence of polarizing cytokines. Th1 conditions indicate addition of IL-12 (10 ng/ml) and anti-IL-4 (10 µg/ml). Th2 conditions indicate addition of IL-4 (10 ng/ml) and anti-IL-12 (10 µg/ml). Th17(β) conditions indicate addition of IL-6 (10 ng/ml), TGFβ-1 (5 ng/ml), anti-IFN-γ (10 µg/ml), and anti-IL-4 (10 µg/ml). Th17(23) conditions indicate addition of IL-6 (10 ng/ml), IL-23 (10 ng/ml), anti-IFN-γ (10 µg/ml), and anti-IL-4 (10 µg/ml). Where indicated, TIMP1 was used at 100 ng/ml. All cytokines were obtained from R&D Systems (Minneapolis, MN). All anti-cytokine antibodies were obtained from BioXCell (West Lebanon, NH). T cells were isolated from the spinal chords of mice with extrinsic allergic alveolitis as follows: The spinal chords were disrupted in cell culture media containing 0.2 mg/ml DNase 1 (Roche, Mannerheim, Germany). Lymphocytes were isolated by centrifugation of the cell suspension in a 40% Percol solution, then analyzed for cytokine expression.

### Measurement of Cytokines

The stimulated naïve cells from above were used for analysis. Detection of IFN-γ-, IL-17-producing cells and FoxP3-expressing cells was determined by intracellular cytokine staining with anti-IFN-γ-APC, anti-IL-17-PE (BD Biosciences, San Jose, CA), or anti-FoxP3-FITC (eBioscience, San Diego, CA). In brief, cells were stimulated for 4 hr with phorbol myristate acetate (50 ng/ml) and ionomycin (500 ng/ml) with Golgistop added after 2 hr. Cell stimulation was terminated by fixing in 4% formyl saline. Fixed cells were stained with fluorescent antibodies in 0.1% saponin permeablization buffer and analyzed on a FACS Calibur flow cytometer (BD Biosciences). Events were collected and analyzed with Flow Jo software (Tree Star Inc, Ashland, OR). Cytokine and secreted protein production in cell culture supernatants was analyzed by enzyme-linked immunosorbent assay (ELISA) with mouse IL-17 Quantikine assay kits (R&D Systems, Minneapolis, MN) and TIMP1 Quantikine assay kits (R&D Systems) according to the manufacturer’s instructions.

### Quantitative Real-time PCR

Total RNA was extracted by RNeasy kit (Qiagen, Valencia, CA). cDNA was synthesized with Reverse Transcription kit (Applied Biosystems, Foster City, CA) using random hexamers as primers according to the manufacturer’s instruction. Actin was used as endogenous control. TaqMan primers and probes for murine TIMP1, IL-17A and GAPDH were purchased from Applied Biosystems, and samples were analyzed using the ABI PRISM 7500 Sequence Detection System (Applied Biosystems).

### Chromatin Immunopreciptation (ChIP)-seq

ChIP-seq experiments were performed as described previously [18]. Briefly, CD4^+^ Th cells were polarized under neutral (Th0), Th1, or Th17 conditions for 72 h. cells were activated for 3 days followed by resting in cytokine-free media for 24 hr followed by the addition of IL-6, IL-12 or IFN-γ for 30 min. DNA-bound transcription factors were subsequently cross-linked by infusing complete medium containing 1% formaldehyde for 15 min followed by sonication of the cell lysate. Chromatin from 2×10^7^ cells was used for each ChIP experiment, which yielded approximately 100 ng of DNA. Antibodies against anti-H3K4Me3, anti-STAT1, anti-STAT3 and anti-STAT4 were used. The ChIP DNA fragments were blunt-ended, ligated to the Illumina adaptors, and sequenced using the Illumina 1G Genome Analyzer. Sequenced reads of 25 bp were obtained using the Illumina Analysis Pipeline (all Illumina, CA). All reads were mapped to the mouse genome (mm9) and only uniquely matching reads were retained. Those unique tags were mapped into non-overlapping 200 bps windows of genome for STAT3 binding sample. Significant peaks (islands) were identified based on window tag-count threshold determined from a *P*-value of 0.05 (defined by Poisson background model). The complete data sets are available at the gene expression omnibus with the following accession code numbers: STAT3 (GSM652873), STAT4 (GSM550303) and STAT1 (GSM994528).

### Gelatin Zymography

Gelatin zymography was used to test whether MMP activity was affected by any of the aforementioned treatment groups. Conditioned medium from each sample was mixed with Tris-Glycine sample buffer, in a ratio of 5∶1, and analyzed by electrophoresis with 10% Novex zymogram gels (Invitrogen) for 120 min. Sample buffer (5x) consisted of 0.313 M TrisHCl, pH 6.8, 10% SDS, 0.05% Bromophenol blue, and 50% glycerol. Recombinant human gelatinase-A was used as a control at a concentration of 1 ng. The gels were developed according to the manufacturer’s instructions, stained using DyeHard Coomassie Blue staining solution (Crystalgen, Commack, NY), and imaged using and Epson Perfection 4490 scanner. Densitometry (ImageQuant v. 5.2) was done, subtracting background and normalizing to the control sample included on each gel.

### Reverse Zymography

Reverse gelatin zymography was used to test whether TIMP1 and TIMP2 activity was affected by any of the treatment groups. The separating gels were prepared with 2.25 mg/ml porcine gelatin (Sigma), 0.25 M Tris-HCl, pH 8.8, 0.125% SDS, 1 µl/ml TEMED, 0.4 mg/ml ammonium persulfate; 15% (w/v) acrylamide and 0.4% bisacrylamide, 128 ng/ml pro-gelatinase A. A standard 5% was used. Conditioned medium from each sample was mixed with Tris-Glycine sample buffer, in a ratio of 5∶1. Recombinant human TIMP1 (R&D Systems) and rhTIMP2 (R&D Systems) were used as a control on each reverse zymogram at concentrations of 10 and 1 ng, respectively. Samples were run at 200 V (constant) for 15 minutes, after which the voltage was dropped to 125 V and the gels were run for an additional 120 minutes. Reverse zymograms were removed and incubated with the same re-naturation and developing reagents used for gelatin zymography (Invitrogen), according to their manufacturer’s instructions. The gels were developed according to the manufacturer’s instructions, stained using DyeHard Coomassie Blue staining solution (Crystalgen), and imaged using and Epson Perfection 4490 scanner. Densitometry (ImageQuant v. 5.2) was done, subtracting background and normalizing to the control sample included on each gel.

### Experimental Autoimmune Encephalomyelitis

EAE was induced as previously described using a passive transfer model [19]. Briefly, splenocytes from 2D2 or *Timp1^−/−^* 2D2 mice were stained with anti-CD4, anti-Vβ11, anti-CD62L and anti-CD44 (BD Bioscience) and naïve (CD4^+^Vβ11^+^CD62L^+^CD44**^−^**) T cells were isolated by flow cytometry cell sorting. The isolated cells were activated for three days under Th1, Th17(β) or Th17(23) conditions. 1×10^6^ activated T cells were injected intro-peritoneally into Rag2^−/−^ host animals. The severity of neurological disease was monitored and evaluated on a scale from 0 to 5 according to Hooke Laboratories guideline: 0 = no disease; 1 = paralyzed tail; 2–3 = one to both hind limbs affected; 4 = hind limbs and a single fore limb affected; 5 = moribund with all limbs affected or found dead. Mice with scores of 4 or greater were euthanized and their last score was entered for the rest of the experiment.

### Statistical Methods

Unless stated otherwise, all statistical comparisons were made using a two-tailed unpaired t-test. Statistical analysis was computed using Prism v5.0 (Graphpad) *P<0.05, **P<0.01.

## Results

### TIMP1 is Selectively Expressed in Th17 Cells

**Figure 1:**
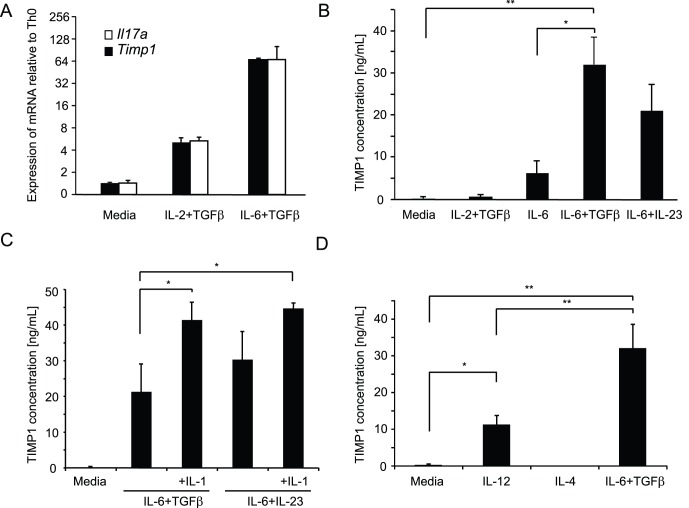
TIMP1 is expressed in T cells activated under Th17 conditions. (**A**) Naïve CD4^+^ T cells were stimulated for three days in media alone, iTreg conditions or Th17(β) conditions. Subsequently, mRNA was isolated and *Il17a* and *Timp1* mRNA analysed by quantitative-PCR. (**B**) Naïve CD4^+^ T cells were stimulated in media alone, IL-6 alone, iTreg conditions, Th17(β) conditions or Th17(23) conditions. After three days, secreted TIMP1 was measured by ELISA. (**C**) Naïve CD4^+^ T cells were polyclonally stimulated in media alone (Th0) or in either Th17(β) conditions or Th17(23) conditions in the presence or absence of IL-1β. After three days, secreted TIMP1 was measured by ELISA. (**D**) Naïve CD4^+^ T cells were stimulated in media alone or under Th1 conditions, Th2 conditions or Th17(β) conditions. After three days, secreted TIMP1 was measured by ELISA. Histograms represent mean values (n = 3 per group), error bars represent s.d. Data are representative of two independent experiments.

To understand the range of genes expressed in Th17 cells that might contribute to their function, we assessed the transcriptome of naïve CD4^+^ T cells stimulated for three days in the absence of cytokines (Th0) or in the presence of TGFβ-1 and IL-2, cytokines that induce T regulatory cells (iTreg) or in the presence of TGFβ-1 and IL-6, cytokines that induce Th17 cells [20]. This study identified a number of genes that were not previously known to be associated with Th17 cells, including the secreted protein tissue inhibitor matrix metalloproteinase 1 (TIMP1). To confirm the preferential expression of *Timp1* mRNA in Th17 cells versus iTreg and Th0 cells, we analyzed expression of *Timp1* mRNA by quantitative-PCR and compared this with the expression of *Il17a* mRNA (**Figure 1A**). These data suggested a close correlation between the expression of the two genes.

Naïve CD4^+^ T cells can differentiate into Th17 cells in vitro through stimulation in the presence of either IL-6 and TGFβ-1 (Th17(β) cells) [21] or IL-6 and IL-23 (Th17(23) cells) [19]. We next explored which Th17 polarizing cytokines were critical for inducing TIMP1 protein by measuring its secretion into the cell culture supernatant by ELISA in cells stimulated in the presence of IL-6 alone or in combination with TGFβ-1 or IL-23 and compared this with Th0 and iTreg cells (**Figure 1B**). In confirmation of the mRNA data, no secreted TIMP1 was detected in the cell culture supernatants containing naïve CD4^+^ T cells stimulated under Th0 and iTreg conditions. In contrast, T cells stimulated under both Th17 conditions secreted high levels of TIMP1. Interestingly, even IL-6 alone was able to induce a modest secretion of TIMP1. IL-1 is an important regulator of IL-17 production [22], [23]. To explore its effect on TIMP1 expression, we stimulated naïve T cells under Th17 conditions in the absence or presence of IL-1 and measured TIMP1 secretion by ELISA. The data show that under both conditions IL-1 induced a significant increase in TIMP1 secretion (**Figure 1C**).

We next sought to assess whether the expression of TIMP1 in Th17 was a unique or shared characteristic with Th1 or Th2 cells. We stimulated naïve CD4^+^ T cells in the presence of IL-12 (Th1 conditions), IL-4 (Th2 conditions) or TGFβ-1 and IL-6 and measured secreted TIMP1 by ELISA. As shown in **Figure 1D**, CD4^+^ T cells polarized under Th1 or Th17 conditions secreted TIMP1 although Th17 cells produce significantly higher levels. In contrast, cells stimulated under Th2 conditions failed to secrete TIMP1.

### Th1 and Th17 Derived TIMP1 is Biologically Active

**Figure 2:**
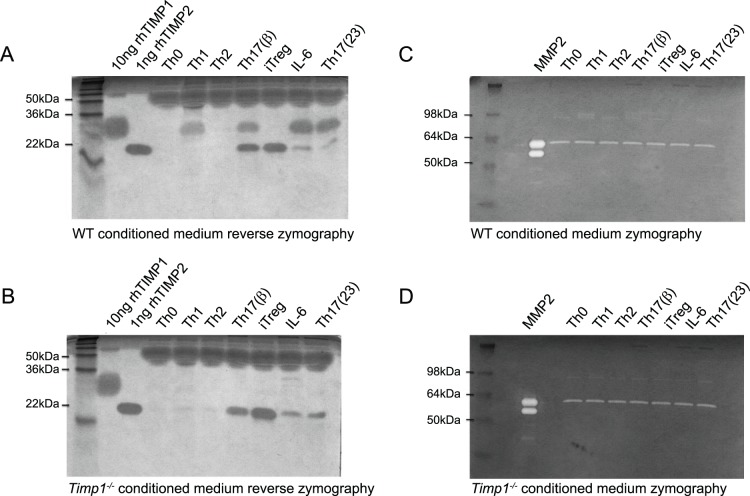
Activated T cells secrete active TIMP proteins by Zymography. Naïve CD4^+^ wild type B6 T cells (**A**) or *Timp1^−/−^* T cells (**B**) were stimulated either under Th0 (media alone), IL-6 alone, Th1 conditions, Th2 conditions, iTreg conditions, Th17(β) or Th17(23) conditions. After three days, samples of supernatant were run on a protein gel together with control samples of TIMP1 and TIMP2. TIMP activity was determined by the ability of the protein gel to resist digestion by metaloprotease. Naïve CD4^+^ wild type B6 T cells (**C**) or *Timp1^−/−^* T cells (**D**) were stimulated either under Th0 (media alone), IL-6 alone, Th1 conditions, Th2 conditions, iTreg conditions, Th17(β) or Th17(23) conditions. After three days, samples of supernatant were run on a protein gel together with control samples of MMP9. MMP9 activity was determined by its ability to digest protein within the gel. Results are representative of two independent experiments.

Despite demonstrating that Th1 and Th17 cells were able to secrete TIMP1 as measured by ELISA, we wanted to ascertain that material secreted was functionally active. To assess this, we used reverse zymography to assess the biological activity of T cell derived TIMP1 and conventional zymography to assess T cell production of MMP2, a known target of TIMP1. Naïve CD4^+^ T cells were polyclonally stimulated under Th0, Th1, Th2, Th17 or iTreg polarizing conditions or in the presence of either IL-6 alone. After three days the supernatants were collected and run on an acrylamide gel containing porcine gelatin. Human TIMP1 and TIMP2 were added as controls to the first two lanes. T cell-derived TIMP1 and TIMP2 were identified by their ability to attenuate gelatin degradation. The experiment indicated in **Figure 2A** shows that activity corresponding to both TIMP1 and TIMP2 was identified in supernatants of Th17 cells. Supernatants from iTreg cells lacked expression of TIMP1 as expected; however, activity corresponding to TIMP2 was detectable. This suggests that TGFβ-1 may be the predominant factor that coordinately regulates expression of TIMP2 in both Th17 and iTreg cells. Consistent with this view, little TIMP2 activity was evident in supernatants from cells treated with IL-12 (Th1) or IL-6. The experiment was repeated using supernatants from *Timp1* deficient mouse T cell cultures to document specificity (**Figure 2B**). We next used conventional zymography to show that T cells secreted similar amounts of MMP2 irrespective of the culture conditions used (**Figure 2C and 2D**).

### TIMP1 is Regulated in Th1 and Th17 Cells by Distinct Mechanisms

The Th17 associated cytokines, IL-6 and IL-23, are potent activators of STAT3, which in turn is necessary for Th17 differentiation in mouse and humans [24], [25]. We therefore next investigated whether TIMP1 induction, like IL-17 itself, is dependent upon STAT3 signaling. To this end, we cultured naïve T cells from control (*Stat3^fl/fl^*) and *CD4-Cre;Stat3^fl/fl^* mice under Th0 or Th17 conditions and measured *Timp1 and Il17a* mRNA by quantitative-PCR. The data in **Figure S1** demonstrates that both *Timp1* and *Il17a* mRNA were highly induced under Th17 conditions in *Stat3^fl/fl^* T cells but not in T cells deficient in STAT3.

**Figure 3:**
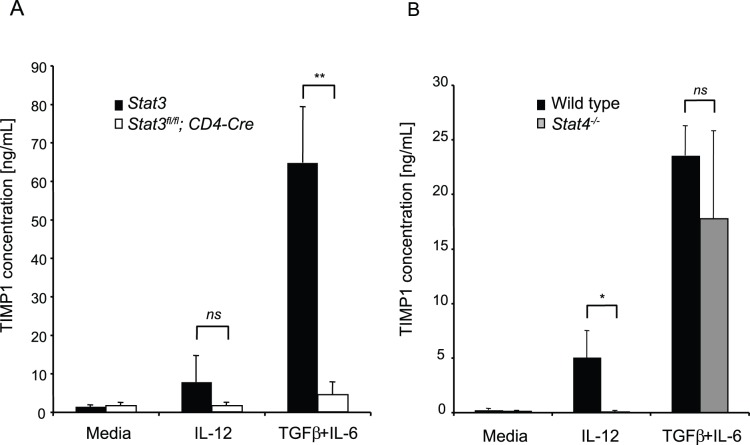
TIMP1 is induced in Th1 and Th17 cells by different mechanisms. (**A**) Naïve CD4^+^ T cells from *Stat3^fl/fl^* (black bars) or *Stat3^fl/fl^;CD4-Cre* (white bars) mice were stimulated in media alone (Th0), Th1 conditions or Th17(β) conditions. After three days, secreted TIMP1 was measured by ELISA. (**B**) Naïve CD4^+^ T cells from wild type (black bars) or *Stat4^−/−^* (grey bars) were stimulated in media alone (Th0), Th1 conditions or Th17(β) conditions. After three days secreted TIMP1 was measured by ELISA. Histograms represent mean values (n = 3 per group), error bars represent s.d. Data are representative of two independent experiments.

Unlike IL-6 and IL-23, IL-12 principally induces the phosphorylation and activation of STAT4, although all three cytokines have the capacity to phosphorylate STAT3. We therefore compared the ability of T cells deficient in either STAT4 or STAT3 to secrete TIMP1 under Th1 or Th17 conditions. The data in **Figure 3A** demonstrates that STAT3-deficient T cells secrete reduced amounts of TIMP1 under either Th1 or Th17 conditions. By contrast, STAT4 is dispensable for induction of TIMP1 under Th17 conditions, but is essential for TIMP1 induction when cells are stimulated with IL-12 (**Figure 3B**).

### Both STAT4 and STAT3 Bind the *Timp1* Gene

**Figure 4:**
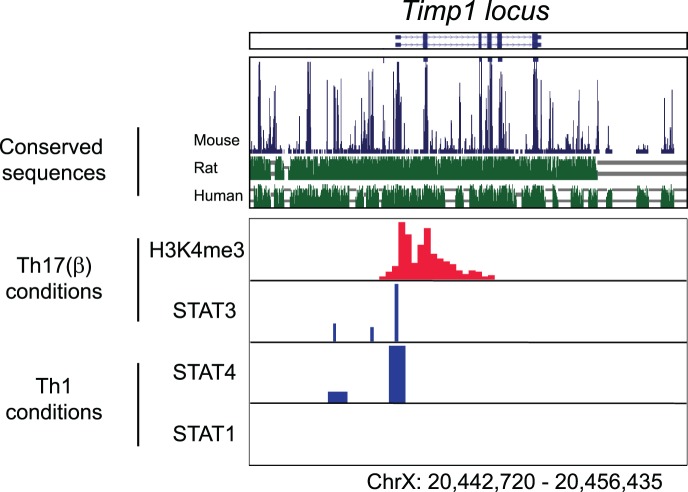
STAT3 and STAT4 are able to bind to the *Timp1* promoter site. Chromatin signatures as defined by the presence of H3K4Me3 or STAT transcription factor binding at the Timp1 gene locus, illustrated at the top of the figure. Th17(β) polarized cells were re-stimulated with IL-6 and immunoprecipitated with anti-H3K4Me3 or STAT3 **(upper two panels).** The STAT3 dataset is taken from [47], the H3K4Me3 dataset is representative of two independent experiments**.** Th1 polarised cells were re-stimulated with CD3/CD28 and IL-12, immunoprecipitated with anti-STAT4 or with anti-STAT1 **(lower two panels).** The STAT4 dataset is taken from [18] and the STAT1 dataset is taken from [48].

The *Timp1* gene resides on the X chromosome within the first intron of synapsin 1. To better understand the means by which STAT3 and STAT4 induce *Timp1* transcription we analysed binding of these transcription factors to the *Timp1* locus using chromatin immuno-precipitation and massive parallel sequencing (ChIP-seq) (**Figure 4** **)** [18]. In the top two panels, naïve CD4^+^ T cells were polarized under Th17 conditions and chromatin immunoprecipitated for STAT3 and Histone3 lysine4 trimethylation (H3K4Me3), a mark that is indicative of the gene locus being open for gene transcription. In the lower two panels, naïve CD4^+^ T cells were polarized under Th1 conditions and subjected to chromatin immunoprecipitation for STAT1 and STAT4. In summary, our findings show STAT3 binding to the *Timp1* locus in cells stimulated in the presence of IL-6 and STAT4 binding to the *Timp1* locus in cells stimulated in the presence of IL-12.

### *Timp1* Deficient 2D2 Th1 and Th17 Cells have Impaired Pathogenicity

**Figure 5:**
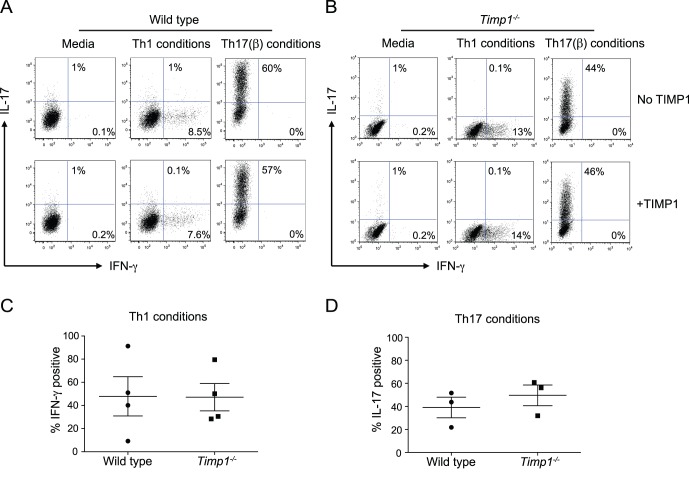
TIMP1 does no alter T cell cytokine expression *in vitro*. Naïve CD4^+^ T cells from wild type (**A**) or *Timp1^−/−^* (**B**) mice were stimulated under Th0, Th1 or Th17(β) conditions in the presence or absence of TIMP1. After three days, cells were fixed and assessed for IFN-γ and IL-17 expression. Naïve CD4^+^ T cells from wild type or *Timp1^−/−^* mice were stimulated under Th1 (**C**) or Th17(β) (**D**) conditions for three days. The percentage IFN-γ^+^ (**C**) or IL-17^+^ (**D**) cells were determined by intracellular staining. The histograms indicate mean ±s.e.m and the data were obtained from four independent experiments.

We were next interested in ascertaining whether TIMP1 was important for the ability of Th1 and Th17 polarized cells to mediate autoimmune disease. In our hands wild type or *Timp1^−/−^* naïve T cells were able to express similar amounts of IFN-γ and IL-17 in the presence or absence of exogenous TIMP1 when stimulated under Th0, Th1 or Th17 conditions respectively (**Figure 5**).

**Figure 6:**
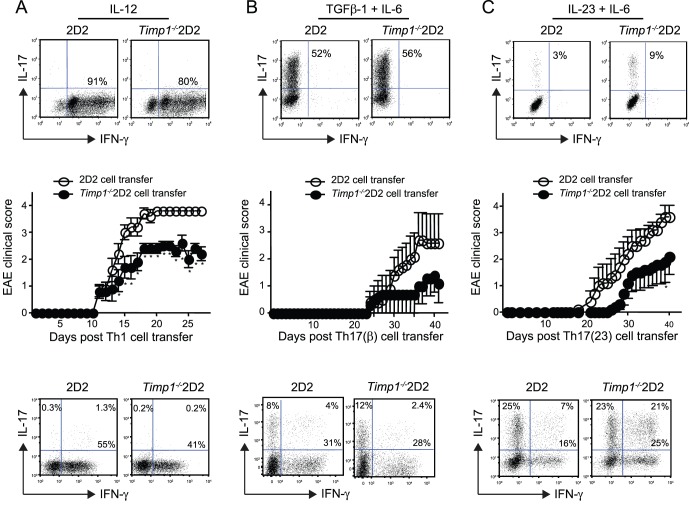
Mice receiving *Timp1* deficient auto-reactive Th1 or Th17 cells are partially resistant to the development of EAE. Naïve CD4^+^ T cells from 2D2 or *Timp1^−/−^*2D2 mice were stimulated under Th1 conditions (**A**), Th17(β) conditions (**B**) or Th17(23) conditions (**C**). After three days cells were fixed and assessed for IFN-γ and IL-17 expression (top panels). Next 10^6^ polarized cells from each group were adoptively transferred into *Rag2^−/−^* recipients, which were followed for signs of neurological disease. Data show mean ±s.e.m of the EAE clinical score of 5 mice per group. After 20 days (Mice receiving Th1 cells) or 35 days (Mice receiving Th17 cells), lymphocytes were isolated from the spinal chords from animals with a clinical score of 3.5 and IL-17 and IFN-γ expression were determined by intracellular staining (lower panels). Significance was determined by a Mann-Whitney U test, *P<0.05. Data are representative of two independent experiments.

Many cells in addition to Th cells secrete TIMP1. To explore the functional significance of T cell derived TIMP1, we performed a passive transfer model of experimental autoimmune encephalomyelitis (EAE) [19]. *Timp1^−/−^* mice were crossed with 2D2 T cell receptor transgenic animals [26]. Naïve CD4^+^ T cells from 2D2 and *Timp1^−/−^*2D2 mice were stimulated in the presence of IL-12 (Th1 conditions), IL-6 and TGFβ-1 (Th17(β) conditions) or IL-6 and IL-23 (Th17(23) conditions). In all cases there was no significant difference in the proportion of cells that secreted either IFN-γ or IL-17 (**Figure 6** **,** top panels).

The remaining stimulated cells were next transferred into *Rag2^−/−^* host animals and the onset of EAE was determined. Clinical scores in Rag2^−/−^ host animals that received Th1, Th17(β) and Th17(23) cells are recorded in **Figures 6A, 6B and 6C** respectively.

In all cases mice receiving *Timp1^−/−^*2D2 cells had both delayed and less severe disease compared with mice receiving 2D2 cells irrespective of how the T cells were stimulated before their transfer into *Rag2^−/−^* host animals although the differences were not significant in mice that received Th17(β) cells. Next we assessed cytokine expression in lymphocytes isolated from the spinal chords of animals, which had received 2D2 or *Timp1^−/−^*2D2 cells with similar disease scores. In keeping with earlier findings we found no difference in the proportion of cells that expressed IFN-γ or IL-17 (**Figure 6** **,** lower panels) suggesting that TIMP1 did not act by altering cytokine expression.

## Discussion

TIMP1 has been implicated in the patho-physiology of a number of inflammatory diseases including psoriasis and rheumatoid arthritis. Although TIMP1 has been found to be expressed within activated T cells [27], [28], we show that CD4^+^ T cells stimulated in the presence of Th1 or Th17 polarizing cytokines up-regulate their expression of TIMP1 mRNA and secrete functional TIMP1 protein as determined by ELISA and reverse zymography. We found that this process was dependent on the activation of STAT3 in both Th17 and to a lesser extent Th1 cells and dependent on STAT4 in Th1 cells. Using ChIP-seq we found that both transcription factors could bind the *Timp1* genetic locus. Finally we demonstrate that autoreactive T cell receptor transgenic *Timp1^−/−^* T cells polarized under Th1, Th17(β) or Th17(23) conditions have an impaired ability to induce EAE. Although the difference was only significant in Th1 and Th17(23) cells. The lack of a significant difference seen in Th17(β) cells may be due in part to the fact that these cells are weakly pathogenic and cause a less severed disease compared with Th17(23) cells [19]. Our findings suggest that T cell derived TIMP1 plays a role in the development of cerebral pathology during EAE.

The matrix metalloproteinases (MMP’s) are the principal enzymes that degrade components of the extracellular matrix [29]. They are implicated in a wide variety of disease processes including tumor metastasis, and joint destruction in rheumatoid arthritis. The TIMP proteins were first discovered as secreted inhibitors of MMP’s. TIMP1 is known to inhibit MMP2 and MMP9. Serum TIMP1 levels are known to be elevated in a number of arthitidites, inflammatory bowel diseases, systemic sclerosis, asthma, Parkinson’s disease and multiple sclerosis [30]–[35]. The precise role of TIMP1 in the patho-physiology of these diseases is not fully understood, in all cases the authors have assumed that TIMP1 is playing a protective role although there is little evidence to support this. The inhibition of MMP’s by TIMP1 should theoretically reduce destruction of the inflamed joint and yet disease severity in Rheumatoid arthritis positively correlates with serum TIMP1 concentration [32]. In oncology TIMP1 has been assumed to play a protective role in inhibiting MMP mediated angiogenesis and metastasis, yet TIMP1 expression in many tumors is associated with a poor prognosis [36], [37].

In our hands we were expecting that *Timp1^−/−^* T cells would cause enhanced disease as MMP2 and MMP9 have been implicated in disrupting the blood brain barrier [38] and mice with forced expression of TIMP1 are resistant to the development of EAE [39]. However, we found that the opposite was true. It is important to note that in our system only T cells were deficient in their ability to secrete TIMP1 and that TIMP1 can potentially have positive and negative roles in the progression of EAE depending on which cell type is either producing or being stimulated by TIMP1.

Our finding that TIMP1 enhances the pathogenic potential of auto reactive T cells in EAE is consistent with the finding that its expression is restricted to Th1 and Th17 cells, the two lineages most associated with the development of EAE. However, the mechanism by which T cell derived TIMP1 could exacerbate EAE is not clear. T cells deficient in TIMP1 demonstrated no differences in cytokine production both before transfer into *Rag2^−/−^* host animals or when isolated from the CNS in animals with similar disease severity (**Fig. 6** lower panels). TIMP1 has other properties in addition to the inhibition of MMP2 and MMP9. TIMP1 is able to bind the intergrin receptor complexes CD63-β1 and αVβ3, in both cases activating phospho-inositol-3-kinase and MAPK signaling leading to stimulation of growth and resistance to apoptosis in the target cell [40]. In this regard, it has been proposed that TIMP1 can be seen as a cytokine with MMP2 and MMP9 as its functional inhibitor [41]. Target cells of TIMP1/intergrin signaling include fibroblasts leading to the promotion of the deposition of matrix proteins and fibrosis. In both Crohn’s disease and systemic sclerosis, TIMP1 has been implicated in mediating excessive fibrosis [31], [42]. The presence of T cell derived TIMP1 may enhance the survival of inflammatory cells during the development of EAE although we were unable to detect significant differences within the inflammatory infiltrate of mice with similar EAE scores (**Fig. 6** lower panels).

Although the selective production of cytokines by helper T cell subsets is a useful concept, it is also increasingly recognized that the distinction between Th1 and Th17 is more blurred than initially envisioned. Th17 cells have a propensity to become IFN-γ producers with multiple rounds of stimulation in vitro [43]. Consistent with this, it has been appreciated that in many autoimmune diseases there is a mixed picture with cells that produce both IL-17 and IFN-γ [19], [44]–[46]. It is therefore useful to consider shared functions of Th1 and Th17 and to consider shared properties that might relate to these functions. The correlation between TIMP1 concentration and disease severity in patients with a variety of autoimmune diseases has lead most researchers to conclude that the presence of TIMP1 is acting to limit inflammation. Our conclusion is that TIMP1 may be acting in a less benign way, analogous to its role in cancer. Thus, a novel array of secreted proteins may contribute to the functional differences between the T helper cell lineages.

## Supporting Information

Figure S1 **Naïve (CD62L^+^ CD44^−^) CD4^+^ T cells from control (** ***Stat3^fl/fl^*** **) and** ***CD4-Cre;Stat3^fl/fl^*** **mice were stimulated under Th0 or Th17(β) conditions.** After three days *Timp1 and Il17a* mRNA were measured by quantitative-PCR. mRNA expression is expressed as fold change compared with T cells stimulated under Th0 conditions.
(EPS)
